# Screening of biomarkers related to lung adenocarcinoma based on construction of ceRNA regulation network

**DOI:** 10.1097/MD.0000000000048837

**Published:** 2026-06-26

**Authors:** Chongyang Niu, Xiaoyu Meng, Chengbo Yuan, Xuelian Sun, Tan Wang

**Affiliations:** a Changchun University of Chinese Medicine, Changchun, Jilin Province, China; b Lung Disease Center, The Affiliated Hospital of Changchun University of Chinese Medicine, Changchun, Jilin Province, China; c Pediatric Center, The Third Affiliated Hospital of Changchun University Chinese Medicine, Changchun, Jilin Province, China.

**Keywords:** ceRNA network, lung adenocarcinoma, prognosis

## Abstract

**Background:**

Lung adenocarcinoma (LUAD) is a common malignant tumor with a poor prognosis and limited effective therapeutic targets. The underlying molecular regulatory mechanisms driving its progression remain largely unclear. The study objectives were to build a circRNA–miRNA–mRNA ceRNA regulation network of LUAD and to identify miRNAs and mRNAs significantly related to the prognosis .

**Methods:**

The gene expression data and GSE101684 were downloaded from the UCSC Xene and NCBI-GEO databases, respectively. The differentially expressed RNAs (DEcircRNAs, DEmiRNAs, and DEmRNAs; DERs) were obtained by the Limma package in R. Then, the differential LUAD-related genes were identified, and the Gene Ontology (GO) and Kyoto Encyclopedia of Genes and Genomes (KEGG) pathways of the differential LUAD-related genes were analyzed. Moreover, the circRNA–miRNA–mRNA ceRNA network of LUAD was built. The Kaplan–Meier (K–M) survival curve analysis of ceRNA network nodes was performed. In addition, the proliferation-related ceRNA network was built.

**Results:**

A total of 382 DEcircRNAs, 1907 DEmRNAs and 156 DEmiRNAs were acquired. A total of 245 differential LUAD-related genes were acquired, which were significantly associated with 189 GO biological processes (BP) and 17 KEGG pathways. Moreover, the ceRNA network of LUAD was built. The K–M survival curve analysis of ceRNA network nodes revealed that a total of 2 miRNAs (hsa-miR-96-5p and hsa-miR-125b-2-3p) and 22 mRNAs (CGNL1, CTHRC1, TK1, etc) were significantly related to the prognosis. mRNAs were significantly enriched in 92 GO BPs (such as cell division, cell adhesion) and 9 KEGG pathways (such as cell cycle, HTLV-1 infection). In addition, the proliferation-related ceRNA network was built.

**Conclusion:**

This research built a ceRNA regulation network of LUAD and is of great significance for identifying biomarkers related to the prognosis in LUAD.

## 1. Introduction

Lung cancer is the most universal malignant tumor, with over 1700 million people diagnosed with lung cancer every year.^[1,2]^ Lung adenocarcinoma (LUAD) is the most universal type of lung cancer. Although the treatment of LUAD has been improved to a large extent, its therapeutic effect is still not very satisfactory.^[3,4]^ Although the treatment of LUAD has been improved to a large extent, its therapeutic effect is still not very satisfactory. The early symptoms of LUAD are usually mild, even without causing any discomfort, so patients with LUAD are usually not detected in the early stage. Therefore, the exploration of characteristic biomolecules significantly associated with LUAD has a crucial role in improving patients’ long-term survival. Thus, the study of non-coding RNAs based on ceRNA network will contribute to find the new prognostic biomarkers of LUAD. In recent years, circular RNA (circRNA) has been identified on some genes, which is considered to be the product of reverse splicing.^[5]^ CircRNAs have many functions connected with tumor progression. A lot of studies have shown that circRNAs can serve as a competitive endogenous RNA (ceRNA) to exert their biological effect.^[6,7]^ Moreover, research has found that circRNA is connected with the pathological process of tumors and is of great significance to the diagnosis of many tumors, such as breast tumors,^[8]^ cervical tumor^[9]^ and prostate tumors.^[10]^ At present, there have been reports on the relationship between long non-codingRNA (lncRNA) and LUAD; however, the circRNA-associated ceRNA network in LUAD progression remains poorly characterized. Thus, a deeper investigation into the ceRNA-based regulatory mechanisms could help us better understand the fine-grained regulatory mechanisms in the occurrence and development of LUAD.

At this research, the ceRNA network of LUAD was built, and we performed the Gene Ontology (GO) and Kyoto Encyclopedia of Genes and Genomes (KEGG) analyses of the ceRNA network. Moreover, the Kaplan–Meier (K–M) survival curve analysis of ceRNA network nodes was performed, and our results found that a total of 2 miRNAs (hsa-miR-96-5p, hsa-miR-125b-2-3p) and 22 mRNAs (CGNL1, CTHRC1, TK1, etc) were significantly related to prognosis. In addition, the proliferation-related ceRNA network was built. All these results help us to better understand the regulatory mechanisms of LUAD.

## 2. Materials and methods

### 2.1. Data collection

The RNA sequencing expression profile data of LUAD was downloaded from the UCSC Xena (https://xenabrowser.net/datapages/) database.^[11]^ This part of the data is log_2_(fragments per kilobase per million [FPKM] + 1). In accordance with the TCGA data annotation criteria, tumor samples were defined as those with sample IDs containing suffixes “-01” (primary tumor), “-02” (recurrent tumor), or “-03” (secondary tumor), whereas para-tumor samples were restricted to those with the suffix “-11” (solid tissue normal). Among them, we regarded the tissues with “-01” in the sample number as cancerous tissues and the tissues with “-11” as paracancerous tissues. Samples with other suffixes (such as “06” and “07,” which denote metastatic tumors or additional new primary tumors, respectively) accounted for <2% of the total dataset. Hence, to ensure the homogeneity of the tumor and para-tumor cohorts, these samples were excluded following careful evaluation. Based on the clinical information of the samples, the cases diagnosed with tumor stages I–II were used as early samples for the next analysis. Finally, a total of 408 cancer tissues and 43 paracancerous tissues were acquired.

In addition, the mature miRNA expression profile data from the UCSC Xena database was downloaded. This part of the data is log_2_(reads per kilobase per million [RPKM] + 1). Among them, we regarded the tissues with “-01” in the sample number as cancer tissues and the tissues with “-11” as the paracancerous tissue, according to the clinical information of the samples, the cases with tumor stage diagnoses as I–II were used as the early samples for the next analysis. Finally, a total of 353 cancer tissues and 39 paracancerous tissues were acquired. Moreover, we downloaded the clinical phenotype data and survival information of the samples.

The GSE101684 (Species: Homo sapiens) dataset was acquired from the Gene Expression Omnibus (GEO, https://www.ncbi.nlm.nih.gov/)^[12]^ based on the platform of the GPL21825 Arraystar Human CircRNA microarray V2.0. The GSE101684 dataset included 8 tumor tissues of patients with early LUAD and corresponding adjacent tissues. The above datasets were downloaded in October 2020.

### 2.2. Data preprocessing

The RNA sequencing data was log_2_(FPKM + 1) data; these genes were annotated according to the gtf gene annotation file (Release 29) provided by GENCODE. The genes whose annotation information was “protein coding” as mRNA were retained, and at least 20% of the mRNAs with expression values >1 for subsequent analysis were reserved. The miRBase database (http://www.mirbase.org/)^[13]^ was used to transform mature miRNA IDs, and at least 20% of the miRNAs with expression values >0 were reserved for subsequent analysis.

### 2.3. Screening of differentially expressed RNAs (DERs)

The Limma package (Version3.34.7, https://bioconductor.org/packages/release/bioc/html/limma.html)^[14]^ was carried out to screen differentially expressed mRNAs (DEmRNAs), miRNAs (DEmiRNAs) and circRNA (DEcircRNAs) of tumor tissues as well as adjacent tissues. All RNAs were analyzed to obtain the corresponding *P* value and logFold Change (FC) values, and the Benjamini & Hochberg method was performed for multiple test correction, and the corrected *P* was adj.*P*, the adj. *P* < .05 and |logFC| > 1 were used as the thresholds.

### 2.4. Further acquisition of LUAD-related genes

Meanwhile, LUAD-related genes were retrieved from the Comparative Toxicogenomics Database http://ctdbase.org/)^[15]^ by searching the keywords “Lung adenocarcinoma,” the genes with inference score ≥15 were selected as LUAD-related genes. The overlapping part of the LUAD-related genes and DEmRNAs obtained in the previous step was retained as the differential LUAD-related genes.

### 2.5. Functional enrichment analysis

The GO and KEGG pathway analyses of the differential LUAD-related genes were performed using DAVID 6.8 (https://davidbioinformatics.nih.gov/),^[16]^ *P* < .05 and enrichment count ≥ 2 were used as screening criteria for significant enrichment results.

### 2.6. Generation of ceRNA network

The Pearson correlation coefficient (PCC) between the differential LUAD-related genes and DEmiRNAs was calculated using the cor.test function in R (version 3.6.1, https://cran-archive.r-project.org/bin/windows/base/old/3.6.1/). The miRNA–mRNA relationship pairs were screened with PCC < −0.4 and adjusted *P* < .05.

#### 2.6.1. *miRNA–mRNA connection relationship construction*

The miRWalk2.0 database (including miRWalk, Microt4, miRanda, PITA, RNA22 and Targetscan; version 2.0, http://mirwalk.umm.uni-heidelberg.de/)^[17]^ was used to search for target genes regulated by miRNA. We chose regulatory relationships that were included in at least 3 of the databases as the miRNA to regulate the mRNA relationship pair. Then, the miRNA–mRNA connection network was built by retaining the intersection with the miRNA–mRNA relationship pairs obtained in the previous section.

#### 2.6.2. *circRNA–miRNA connection relationship construction*

For the DEcircRNAs, we first compared them with the circbase database (http://www.circbase.org/)^[18]^ and retained the circRNAs with the highest score and 100% matching rate, and then converted them into the universal circRNA ID and corresponding sequence. The cirRNA and DEmiRNAs obtained in the previous step were screened by the miranda (v3.3a) software for circRNA–miRNA relationship pairs with Score ≥200 and Energy ≤ −20.

#### 2.6.3. Generation of ceRNA regulatory network

Combining miRNA–mRNA and circRNA–miRNA relationships from (A) and (B), the circRNA–miRNA–mRNA network was built. The CytoNCA plug-in (version 2.1.6, http://chianti.ucsd.edu/cytoscape-3.4.0)^[19]^; Cytoscape (version 3.4.0, /http://apps.cytoscape.org/apps/cytonca) was used to analyze the degree of node connectivity. The parameter was set to without weight, the higher the degree of connectivity, the higher the importance of the node in the network.

### 2.7. *K–M survival curve analysis of ceRNA network*

Based on the clinical information and gene expression data (log_2_(FPKM + 1)) for LUAD, a total of 385 cases with survival information and mRNA expression values, and 341 cases with survival information and miRNA expression values, were finally obtained.

The mRNAs and miRNAs contained in the aforementioned ceRNA network were regarded as candidate RNA molecules. Combining their expression values in the TCGA database with the prognostic information of the samples, and using the median risk score (RS) as the cutoff, the samples were divided into 2 groups (high- and low-risk group) in the TCGA dataset. The association with the prognosis was measured by applying the K–M curve in the R3.6.1 language survival package (version 2.41-1). Meanwhile, the Log-rank test was used to calculate the *P*-value, and RNAs with *P* < .05 were screened.

### 2.8. Function enrichment analysis of mRNA in ceRNA network

The GO and KEGG analyses of the ceRNA regulatory network were carried out through DAVID 6.8 (https://david.ncifcrf.gov/),^[16]^ *P* < .05 and count ≥2 were used as the screening threshold.

## 3. Results

### 3.1. DERs screening

**Figure 1.**
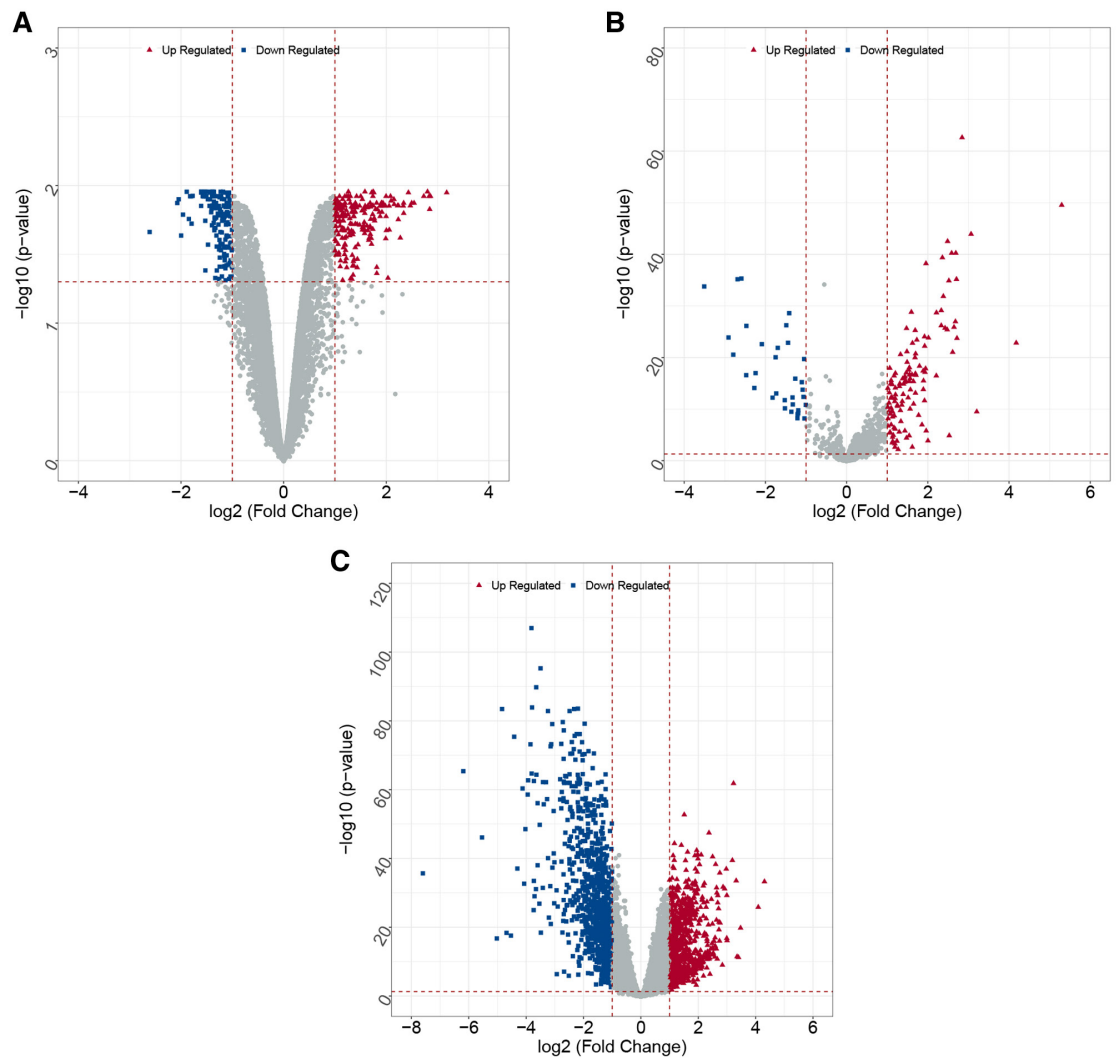
The volcano map of DERs (A: DEmRNAs, B: DEcircRNAs, C: DEmiRNAs). Cutoff criteria were *P* value < .05 and |log_2_FC| > 1. Red and blue dots indicate markedly up-regulated and down-regulated RNAs, respectively, and gray dots represent nonsignificant DERs. DEcircRNAs = differentially expressed circRNA, DEmiRNAs = differentially expressed miRNAs, DEmRNAs = differentially expressed mRNAs, DERs = differentially expressed RNAs, FC = fold change.

**Figure 2.**
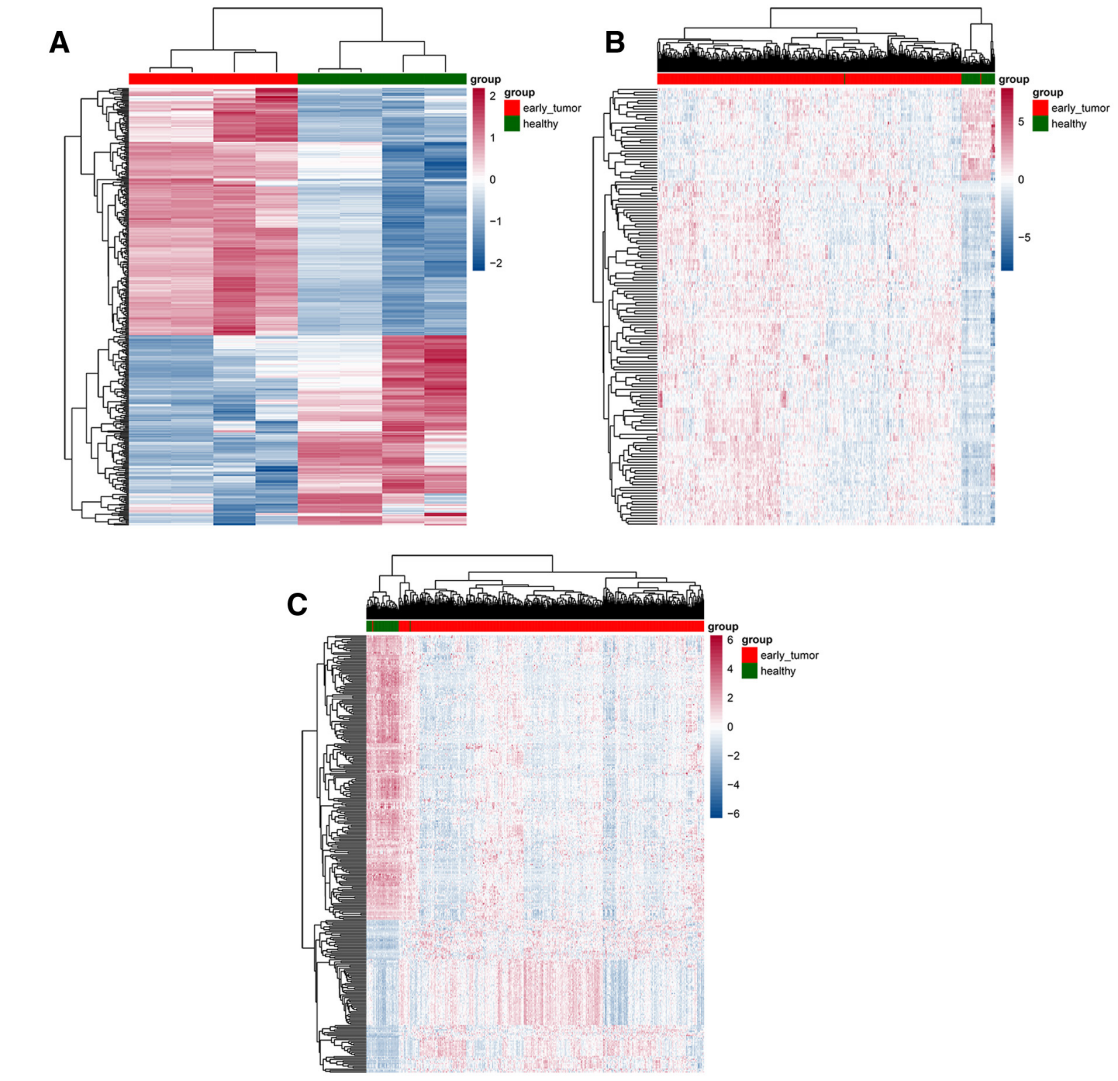
Two-way hierarchical clustering chart based on the expression values of significant DERs from the induction group: (A) DEmRNAs, (B) DEcircRNAs, and (C) DEmiRNAs. Red represents up-regulated DERs, and blue represents down-regulated DERs. Red bars indicate early LUAD tissue, and green bars indicate paracancerous tissue. DEcircRNAs = differentially expressed circRNA, DEmiRNAs = differentially expressed miRNAs, DEmRNAs = differentially expressed mRNAs, DERs = differentially expressed RNAs, LUAD = lung adenocarcinoma.

The differential expression analysis of circRNAs, mRNAs, and miRNAs was performed, and a total of 1907 DEmRNAs, 382 DEcircRNAs, and 156 DEmiRNAs were screened with *P* < .05 and |log_2_FC| > 1 (Table S1, Supplemental Digital Content 1). The DERs are shown in the volcano map (Fig. 1). We displayed the DERs on a heatmap based on the value of |log_2_FC| (Fig. 2). The expression values of DERs were bidirectionally clustered, and the color contrast showed that there was a significant difference in the expression levels between the paracancerous and LUAD cases.

**Figure 3.**
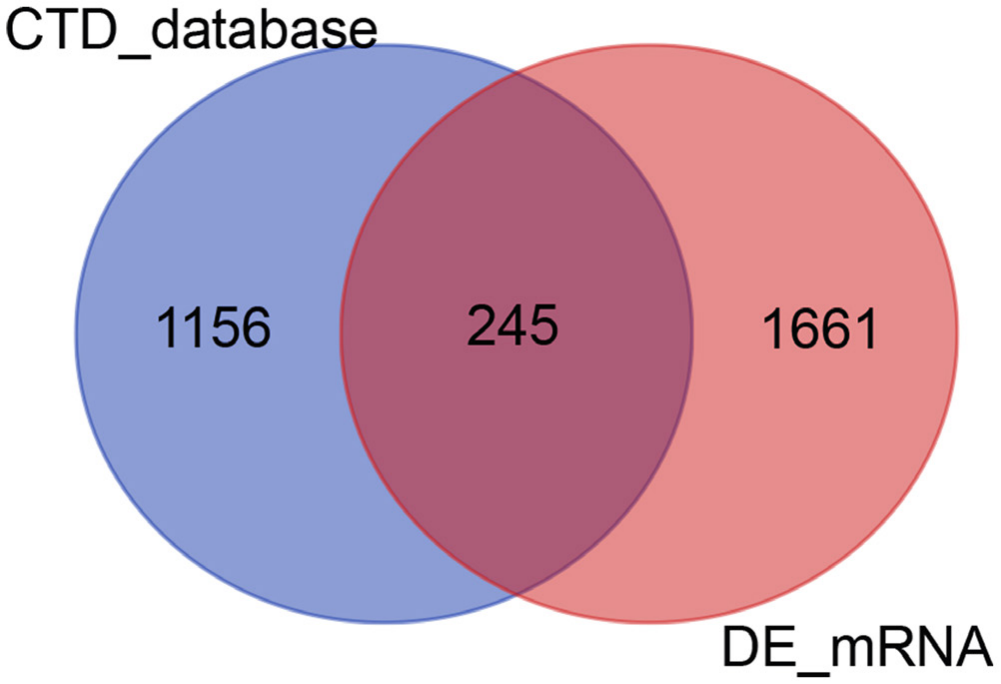
Venn diagram for screening LUAD-related mRNA. LUAD = lung adenocarcinoma.

LUAD-related genes were obtained from the CTD (http://ctdbase.org/) by searching the keywords “Lung adenocarcinoma,” the genes with Inference score ≥15 were selected, and a total of 1401 LUAD-related genes were obtained. After comparing DEmRNAs obtained in the previous steps and LUAD-related genes, a total of 245 overlapping genes were acquired (Fig. 3).

### 3.2. Functional enrichment analysis

**Figure 4.**
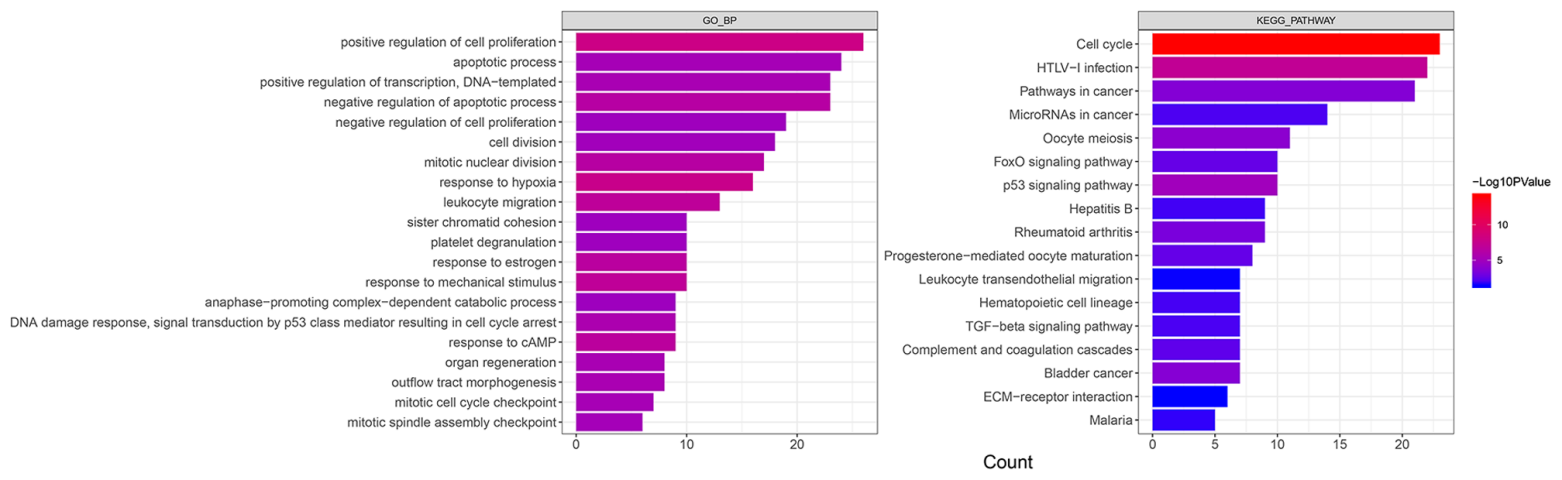
The top 20 GO and KEGG signaling pathway analyses of differentially expressed LUAD-related genes. The horizontal axis represents the gene count, the vertical axis indicates the GO and KEGG entry name, and the color suggests significance; the closer the color is to red, the greater the significance. BP = biological process, GO = Gene Ontology, KEGG = Kyoto Encyclopedia of Genes and Genomes, LUAD = lung adenocarcinoma.

A total of 189 GO BPs and 17 KEGG signaling pathways were obtained through the DAVID tool. Here, we only show the term of top 20 (ranked according to *P*-value). Among them, we found that the differential LUAD-related genes were obviously associated with BPs, such as positive regulation of cell proliferation, response to hypoxia, and leukocyte migration (Fig. 4). Similarly, the differential LUAD-related genes were obviously associated with KEGG signaling pathways, such as cell cycle, HTLV-1 infection, and p53 signaling pathway (Fig. 4).

### 3.3. Generation of ceRNA network

The PCC between the 245 differential LUAD-related genes and 156 DEmiRNAs was calculated. In total, 2737 miRNA–mRNA relationship pairs were obtained with PCC < −0.4 and adjusted *P* < .05.

**Figure 5.**
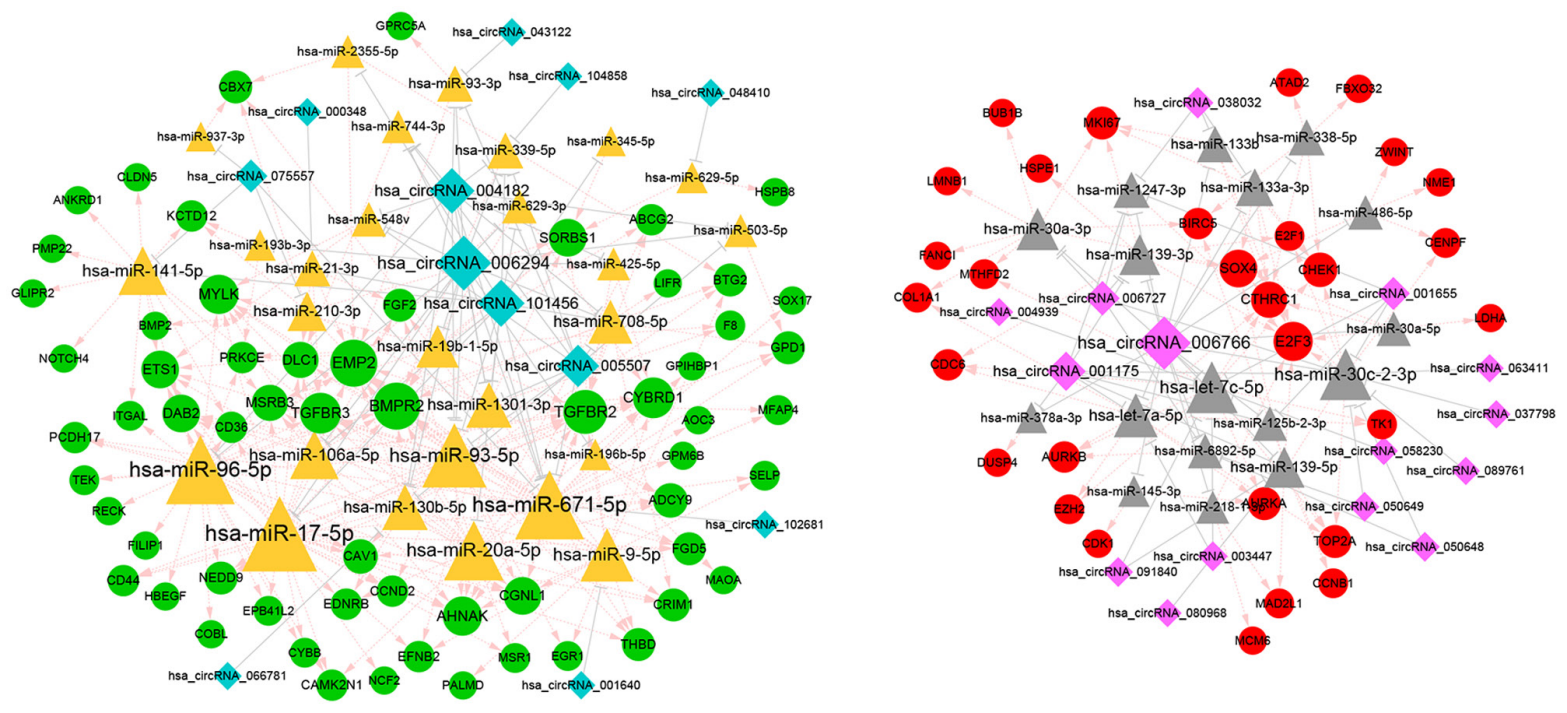
ceRNA regulation connection network. The red and green circles represent up-regulated and down-regulated mRNAs, respectively; the purple and blue diamonds represent up-regulated and down-regulated circRNAs, respectively; the yellow and gray triangles represent up-regulated and down-regulated miRNAs, respectively; the gray T-shaped connection represents circRNA competitive binding of miRNA, and the pink arrow represents the miRNA-mRNA regulatory relationship. ceRNA = competitive endogenous RNA, circRNA = circular RNA.

Seven hundred two circRNA–miRNA connection pairs and 590 miRNA–mRNA pairs were obtained. Based on the circRNA–miRNA and miRNA–mRNA relationship pairs obtained above, first, the circRNA–miRNA–mRNA relationship pairs regulated by the same miRNA were screened. Then, the circRNA–miRNA–mRNA relationship pairs with the same regulatory relationship between mRNAs and circRNAs were obtained. Finally, we obtained 920 circRNA–miRNA–mRNA relationship pairs. The ceRNA network was built as shown in Figure 5, which included 27 circRNAs, 44 miRNAs, and 92 mRNAs. The connectivity degree of each node in the network was analyzed to determine the degree of mRNAs, miRNAs, and circRNAs.

### 3.4. *K–M survival curve analysis of the ceRNA network*

**Figure 6.**
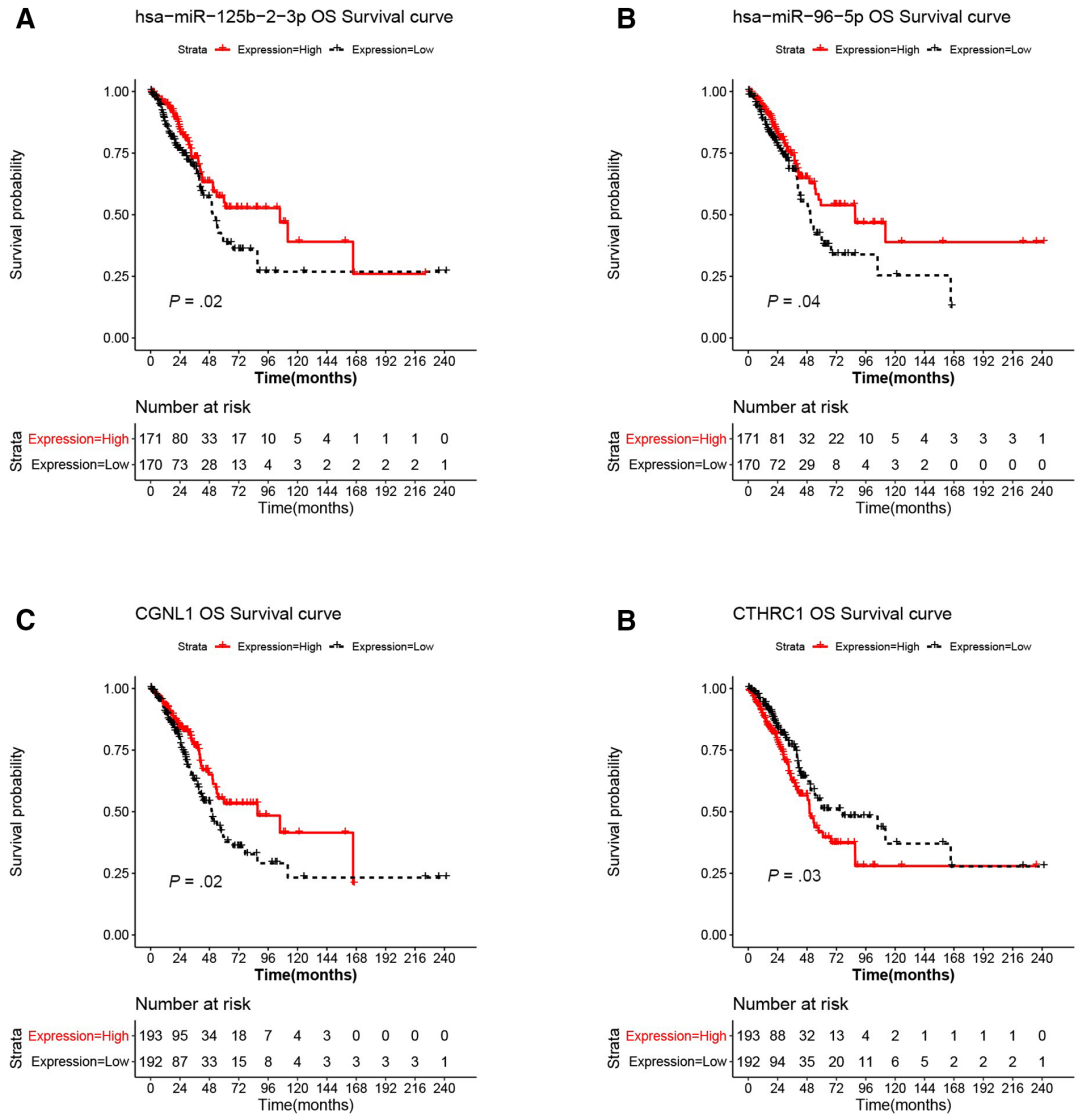
Kaplan-Meier survival curves for overall survival based on the expression of key miRNAs and mRNAs. The black and red curves indicate the low- and high-risk samples, respectively. K–M = Kaplan–Meier.

Combining the survival information of the samples (OS and os.time), the samples were separated into high and low groups based on the median RS expression value of each miRNA and mRNA in the ceRNA network. Moreover, the K–M survival curves were drawn. The results indicated that a total of 2 miRNAs (hsa-miR-96-5p and hsa-miR-125b-2-3p) and 22 mRNAs (CGNL1, CTHRC1, etc) were significantly related to the prognosis. Here we only showed the 2 most connected K–M survival curves (Fig. 6).

### 3.5. Function enrichment analysis of mRNA in ceRNA network

**Figure 7.**
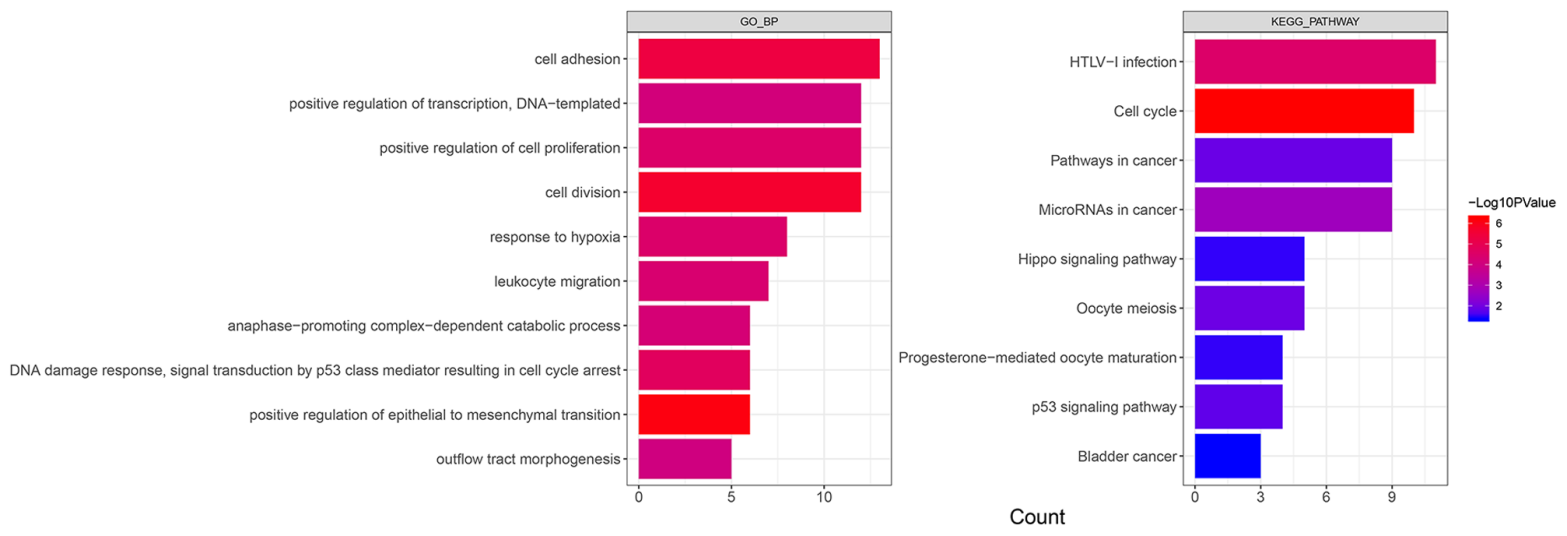
Enrichment analysis of top 10 GO biological processes and KEGG pathways associated with significant genes in the ceRNA regulatory network. The horizontal axis represents the gene count, the vertical axis indicates the GO and KEGG entry name, and the color suggests significance; the closer the color is to red, the greater the significance. BP = biological process, ceRNA = competitive endogenous RNA, GO = Gene Ontology, KEGG = Kyoto Encyclopedia of Genes and Genomes.

The GO and KEGG pathway analysis of DEmRNAs in the ceRNA network was carried out through DAVID software. We acquired a total of 92 GO BPs and 4 KEGG pathways. Here we only showed the term of top 10 (ranked according to *P*-value). Among them, we found that the DEmRNAs were obviously associated with GO terms, such as cell division, cell adhesion and positive regulation of epithelial to mesenchymal transition (Fig. 7). The DEmRNAs were obviously associated with KEGG signaling pathways, such as cell cycle, HTLV-1 infection and microRNAs in cancer (Fig. 7).

### 3.6. Construction of proliferation-related ceRNA network

**Figure 8.**
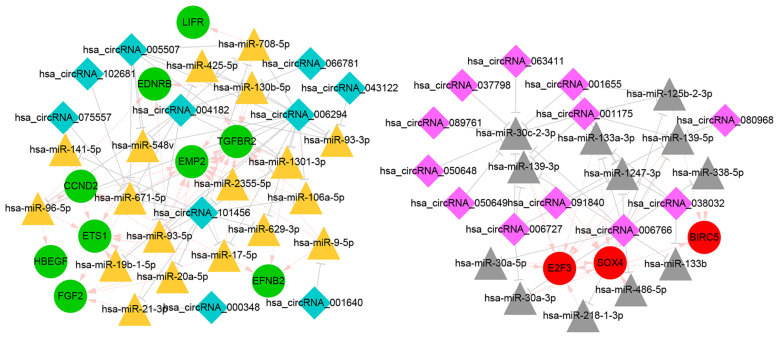
Construction of proliferation-related ceRNA network. The red and green circles represent up-regulated and down-regulated mRNAs, respectively; the purple and blue diamonds represent up-regulated and down-regulated circRNAs, respectively; the yellow and gray triangles represent up-regulated and down-regulated miRNAs, respectively; the gray T-shaped connection represents circRNA competitive binding of miRNA, and the pink arrow represents the miRNA–mRNA regulatory relationship. ceRNA = competitive endogenous RNA, circRNA = circular RNA.

The GO term related to proliferation among the top 10 of the above mRNA enrichment results was selected: GO:0008284 (positive regulation of cell proliferation), and 12 mRNAs involved in the proliferation process were extracted. Furthermore, the corresponding circRNA–miRNA–mRNA relationship was extracted to build a ceRNA network, which was considered to be a ceRNA network related to cell proliferation (Fig. 8).

## 4. Discussion

LUAD has always been regarded as one of the most deadly cancers in adults, causing a huge financial burden every year, especially for advanced LUAD.^[1]^ Although some achievements have been made in the diagnosis and treatment of LUAD, including gene-targeted therapy and immunotherapy, they are not suitable for all patients; some patients may develop drug resistance, or some patients are not sensitive to their therapy.^[3,4]^ Thus, it is important to determine the specific biomarkers of LUAD for early diagnosis and evaluation of patient prognosis.

ceRNA is a novel way to study the interactions of RNAs.^[6]^ Research has proved that ceRNA is linked to the progression of cancers, including breast cancer,^[7]^ glioblastoma,^[20]^ and lung cancer,^[6]^ etc. miRNAs are considered to be a key regulator of gene expression. Studies have found that miRNAs could be regarded as an excellent biomarkers for cancer diagnosis, prediction, and treatment targets, and miRNAs affect the development of cancer.^[21,22]^ Therefore, the identification of important biomarkers is very meaningful for understanding the development of LUAD.

At this research, the bioinformatics analysis was performed to acquire the differential LUAD-related genes, we found that the differential LUAD-related genes were obviously associated with GO, such as positive regulation of cell proliferation, response to hypoxia, and leukocyte migration; the differential LUAD-related genes were obviously associated with KEGG signaling pathways, such as cell cycle, HTLV-1 infection, and p53 signaling pathway. Moreover, we built the ceRNA regulatory network. To understand the relationship between RNAs (miRNAs, mRNAs) and prognosis, we performed K–M survival analysis of each miRNA and mRNA in the ceRNA network. We found that a total of 2 miRNAs (hsa-miR-96-5p and hsa-miR-125b-2-3p) and 22 mRNAs (CGNL1, CTHRC1, TK1, etc) were significantly related to the prognosis. Hsa-miR-96-5p has been confirmed as a key biomarker to study the molecular mechanism of hepatic carcinoma,^[23]^ osteosarcomas with lung metastasis,^[24]^ non-small cell lung cancer,^[25]^ etc. Studies have proved that hsa-miR-96-5p could participate in the PI3K-AKT pathway of colorectal cancer.^[26]^ hsa-miR-125b-2-3p has been confirmed to play a key role in the development of squamous cell carcinoma.^[27]^ It has been proved that hsa-miR-125b-2-3p is significantly related to the prognosis of colon cancer.^[28]^ Research has shown that CGNL1 is associated with the process of renal cell carcinoma,^[29]^ and angiogenesis.^[30]^ CTHRC1 has been proved to play a vital role in renal cell carcinoma,^[31]^ bladder cancer,^[32]^ and papillary thyroid cancer,^[33]^ etc. Meanwhile, GO and KEGG pathway analysis of the ceRNA network was carried out. The GO analysis suggested that the significant genes of the ceRNA regulatory network were mainly involved in cell division, cell adhesion and positive regulation of epithelial to mesenchymal transition, and the KEGG pathway analysis suggested that the significant genes of the ceRNA regulatory network were linked to cell cycle, HTLV-1 infection and microRNAs in cancer.

It is noted that conventional mRNA and lncRNA screening methodologies predominantly concentrate on individual molecular entities and linear regulatory associations.^[34]^ In contrast, the ceRNA network constructed in this study emphasizes the multilevel and interrelated regulatory patterns among miRNAs, lncRNAs and mRNAs. This network-based approach better elucidates the synergistic effects of multiple RNAs in LUAD oncogenesis, while overcoming the limitations of single-molecule screening approaches. Furthermore, unlike conventional approaches that analyze mRNA or lncRNA expressionin isolation, the ceRNA regulatory network framework elucidates the intricate crosstalk among different RNA species, thereby providing a more comprehensive perspective for identifying functional biomarkers.^[35]^ In this study, instead of being obscured by traditional single-RNA profiling techniques, the prognostic markers identified through the ceRNA network not only exhibit significant differential expression levels, but also participate in crucial regulatory pathways. However, while our findings provide a novel insight into the prognosis of LUAD, the study has certain limitations, primarily stemming from its reliance on data obtained from public databases, which may restrict the generalizability of the conclusions. To address this, future research should conduct in vitro and in vivo experiments as well as clinical cohort studies to validate and extend these findings.

## 5. Conclusion

In conclusion, a ceRNA network was successfully constructed for LUAD. Through comprehensive analysis, 2 microRNAs (hsa-miR-96-5p and hsa-miR-125b-2-3p) and 22 messenger RNAs (including CGNL1, CTHRC1, and TK1) were identified as related to prognosis. These findings not only enhance the understanding of the molecular mechanisms driving LUAD tumorigenesis but also offer novel prognostic biomarkers and potential therapeutic targets for this malignancy.

## Author contributions

**Conceptualization:** Chongyang Niu, Tan Wang.

**Data curation:** Chongyang Niu.

**Formal analysis:** Xiaoyu Meng, Chengbo Yuan.

**Investigation:** Xiaoyu Meng, Tan Wang.

**Methodology:** Chongyang Niu, Xiaoyu Meng, Xuelian Sun.

**Software:** Chongyang Niu, Chengbo Yuan.

**Writing – original draft:** Chongyang Niu, Xuelian Sun.

**Writing – review & editing:** Tan Wang.

## Correction

This article was originally published with an incorrect affiliation cue to author Chengbo Yuan. The incorrect affiliation cue has now been changed online from “Chengbo Yuan^c^” to “Chengbo Yuan^b^”


